# Oxidative Stress Triggered by Apigenin Induces Apoptosis in a Comprehensive Panel of Human Cervical Cancer-Derived Cell Lines

**DOI:** 10.1155/2017/1512745

**Published:** 2017-01-16

**Authors:** Raquel P. Souza, Patrícia de S. Bonfim-Mendonça, Fabrícia Gimenes, Bianca A. Ratti, Vanessa Kaplum, Marcos L. Bruschi, Celso V. Nakamura, Sueli O. Silva, Silvya S. Maria-Engler, Marcia E. L. Consolaro

**Affiliations:** ^1^Postgraduate Program in Bioscience and Physiopathology, Universidade Estadual de Maringá (UEM), Av. Colombo 5790, 87025-210 Maringá, PR, Brazil; ^2^Clinical Cytology and STD Laboratory, Department of Clinical Analysis and Biomedicine, Universidade Estadual de Maringá (UEM), Av. Colombo 5790, 87025-210 Maringá, PR, Brazil; ^3^Postgraduate Program in Pharmaceutical Sciences, Laboratory of Research and Development of Drug Delivery Systems, Universidade Estadual de Maringá (UEM), Av. Colombo 5790, 87025-210 Maringá, PR, Brazil; ^4^Postgraduate Program in Pharmaceutical Science-Clinical Analysis Area, Universidade de São Paulo (USP), Av. Lineu Prestes 580, 05508-900 São Paulo, SP, Brazil

## Abstract

Recently, the cytotoxic effects of apigenin (4′,5,7-trihydroxyflavone), particularly its marked inhibition of cancer cell viability both in vitro and in vivo, have attracted the attention of the anticancer drug discovery field. Despite this, there are few studies of apigenin in cervical cancer, and these studies have mostly been conducted using HeLa cells. To evaluate the possibility of apigenin as a new therapeutic candidate for cervical cancer, we evaluated its cytotoxic effects in a comprehensive panel of human cervical cancer-derived cell lines including HeLa (human papillomavirus/HPV 18-positive), SiHa (HPV 16-positive), CaSki (HPV 16 and HPV 18-positive), and C33A (HPV-negative) cells in comparison to a nontumorigenic spontaneously immortalized human epithelial cell line (HaCaT). Our results demonstrated that apigenin had a selective cytotoxic effect and could induce apoptosis in all cervical cancer cell lines which were positively marked with Annexin V, but not in HaCaT (control cells). Additionally, apigenin was able to induce mitochondrial redox impairment, once it increased ROS levels and H_2_O_2_, decreased the Δ*ψm*, and increased LPO. Still, apigenin was able to inhibit migration and invasion of cancer cells. Thus, apigenin appears to be a promising new candidate as an anticancer drug for cervical cancer induced by different HPV genotypes.

## 1. Introduction 

At present, cervical cancer is the fourth leading cause of cancer among women worldwide, despite the existence of highly effective prevention and screening methods [1, 2]. Persistent high-risk human papillomavirus (HR-HPV) infection is the central factor in the development of cervical cancer, and HPV 16 and HPV 18 account for approximately 70% of all cases of this cancer [1–4]. Chemoradiotherapy is a standard treatment option for patients with unresectable and locally advanced cervical cancer [5]. The 5-year survival rate of advanced cervical cancer has significantly improved due to the application of concurrent chemoradiotherapy in recent years. However, local recurrence and distant metastasis are still common posttreatment manifestations in patients with advanced cervical cancer. Once posttreatment failure occurs, prognosis becomes worse: the 1-year survival rates of patients with such failures are less than 20% [6]. Moreover, various side effects are produced that can greatly influence a patient's quality of life [7]. Despite these alarming facts, efficient methods of treatment are still lacking.

**Figure 1:**
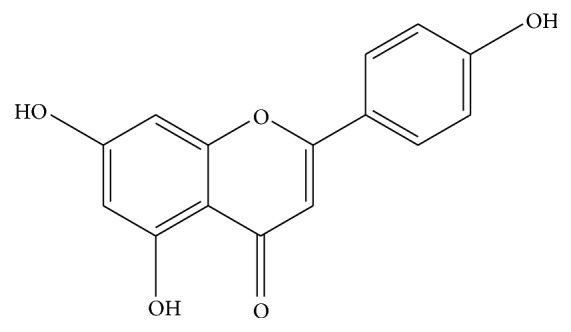
Chemical structure of apigenin.

In recent decades, various natural products have been evaluated as potential anticancer drugs, both in unmodified (naturally occurring) and modified (synthetically modified) forms [8]. Almost 50% of all anticancer agents that have entered clinical use since 1940 are either natural products or their direct derivatives [9]. Flavonoids are a class of plant secondary metabolites that exhibit a variety of activities, including antibacterial, antiviral, antioxidant, and anticancer effects [10]. Flavonoids comprise approximately 6,000 compounds that are characterized and are distinguished from other aromatic compounds by having a common phenylchromanone structure (C6-C3-C6) consisting of two benzene aromatic rings (A and B rings) linked by three carbons that are usually in an oxygenated central pyrone ring (C ring) [11–13]. Based on the saturation level and opening of the central pyran ring, flavonoids can be classified into distinct subclasses including flavanols, flavanones, flavanonols, flavonols, anthocyanidins, isoflavones, and flavones [14–16]. Flavones and flavonols are structurally similar compounds, with flavonols having an extra hydroxyl substitution at the carbon 3-position. Apigenin is a flavonoid belonging to the flavone structural class and chemically known as 4′,5,7,-trihydroxyflavone (Figure 1). Apigenin is a low molecular weight flavonoid (MW 270.24) structurally forming yellow needles in pure form. It is incompatible with strong oxidizing agents [17]. Apigenin exist in propolis as well as in vegetables and fruits such as onions, oranges, and parsley [14, 18]. Apigenin possesses significant actions that suppress inflammation, viruses, oxidation, and carcinogenesis [19].

Apigenin is of particular interest as an antitumor agent since it exhibits lower intrinsic toxicity and is not mutagenic compared to other structurally related flavonoids [20–23]. Furthermore, apigenin has shown marked effects in inhibiting cancer cell growth in cell culture systems and in in vivo tumor models [14, 19] of a variety of human cancers, including colon, breast, pancreatic, oral squamous, lung, ovarian, prostate, and skin cancer as well as leukemia, by regulation of diverse signaling pathways [21, 24–30]. Apigenin has also been demonstrated to inhibit tumor cell invasion and metastasis [19, 31]. According to previous studies, apigenin inhibits insulin-like growth factor 1 (IGF-1) induced cell cycle progression and insulin receptor substrate-1 (IRS-1) tyrosine phosphorylation and upregulates insulin-like growth factor binding protein 3 (IGFBP-3) through modulation of IGF axis signaling in prostate cancer [32, 33]. IGF-1 promotes intercellular signaling through links with the IGF-1 receptor which exists in various primary cells. IGF-1 receptor signaling is associated with activation of phosphatidylinositol 3-kinase (PI3K) and mitogen-activated protein kinase (MAPK) pathways that stimulate proliferation and apoptosis of cells [34]. In addition, apigenin inhibits proliferation of lung cancer cells by inhibiting vascular endothelial growth factor (VEGF) transcriptional activation and by inhibiting the phosphorylation of AKT and P70S6K [35]. Moreover, apigenin suppresses aflatoxin B1 which is the most toxic aflatoxin involved in hepatocellular carcinoma and stimulates cell cycle arrest and reduction of CDK4 with an increase in p53 and p21, respectively, in hepatocellular carcinoma [36–38].

Although apigenin represents a promising chemotherapeutic agent for cancer therapy, little information is available regarding the effects of apigenin against cervical cancer, and the available data were mostly obtained in the HeLa cell line [39–42]. HeLa cells were derived from a case of adenocarcinoma of the uterine cervix in 1952 and contain integrated HPV 18 [43]. Notably, previous studies did not assess the cytotoxic activity of apigenin in cell lines containing HPV 16, which is the most prevalent genotype and the main causative agent of squamous cell cervical cancer (approximately 50% of total) [1, 4, 44]. Additionally, the cytotoxic activity of apigenin has not been evaluated in cell lines derived from squamous cell cervical cancer, which is the most common type of cervical cancer worldwide (75%–85% of total) [45, 46].

In the present study, we investigated the antitumoral effects of apigenin in a comprehensive panel of human cervical cancer-derived cell lines, including HeLa (HPV 18-positive), SiHa (HPV 16-positive), CaSki (HPV 16 and 18-positive), and C33A (HPV-negative), compared to a nontumorigenic human epithelial cell line (HaCaT). The objectives of this study were to investigate effects of apigenin on HeLa, SiHa, CaSki, C33A, and HaCaT cells with respect to (i) cell cytotoxicity, migration, and invasion of those cells; (ii) cell death pathway and cellular oxidative stress. Our results demonstrated that apigenin has a selective cytotoxic effect: it induced apoptosis in all cervical cancer cell lines, but not in HaCaT cells. Additionally, apigenin induced mitochondrial redox impairment and inhibited cancer cell migration and invasion.

## 2. Materials and Methods

### 2.1. Chemicals

Apigenin (4′,5,7-trihydroxyflavone) with ≥ 98% purity was purchased from Cayman Chemical Company (Cat No. 10010275, Ann Arbor, MI, USA). Dimethyl sulfoxide (DMSO), diphenyl-1-pyrenylphosphine (DPPP), Dulbecco's modified Eagle's medium (DMEM), violet crystal, and tetramethylrhodamine ethyl ester (TMRE) were purchased from Sigma-Aldrich (St. Louis, MO, USA). Fetal bovine serum (FBS), penicillin/streptomycin, trypsin/EDTA solution, and trypan blue were purchased from Gibco (Grand Island, NY, USA). Annexin V/FITC, MTT [3-(4,5-dimethylthiazol-2-yl)-2,5-diphenyltetrazolium bromide] and propidium iodide were obtained from Invitrogen (Eugene, OR, USA). X-Gal was obtained from Life Technologies (Grand Island, NY, USA). 2′,7′-Dichlorodihydrofluorescein diacetate (H_2_DCFDA) was obtained from Molecular Probes (Eugene, OR, USA). Matrigel was purchased from Becton–Dickinson (San Jose, CA, USA). All other reagents were purchased from Synth (Diadema, SP, BR).

### 2.2. Cell Lines and Culture Conditions

Human cell lines derived from invasive cervical cancer, including the HeLa (integrated HPV 18), SiHa (1 to 2 copies of HPV 16 integrated per cell), and CaSki (approximately 600 copies of HPV 16 integrated per cell, as well as sequences of HPV 18) lines, as well as the spontaneously immortalized human epithelial cell line HaCaT (nontumorigenic control cells) [47, 48], were kindly donated by Dr. Luisa L. Villa (ICESP, School of Medicine, University of São Paulo/Brazil) and Dr. Silvya S. Maria-Engler (Faculty of Pharmaceutical Sciences, University of São Paulo). The C33A cell line, a human cell line derived from invasive cervical cancer, was purchased from the American Type Culture Collection (Rockville, MD; HTB-31). All cell lines were maintained in a culture flask in DMEM supplemented with 10% fetal bovine serum (FBS) and 0.5 U/mL of penicillin/streptomycin at 37°C in a 5% CO_2_ atmosphere at 100% humidity. HaCaT cells were maintained under the same conditions but were cultured with high-glucose DMEM.

### 2.3. Treatments

Apigenin was dispersed in dimethyl sulfoxide (DMSO) at a concentration of 200 mM and stored at −20°C. After reaching subconfluence (70%–80% confluency), cells were exposed to apigenin diluted in DMEM (2.5–100 *μ*M) [40, 41] for 24, 48, and 72 h. Cells treated with DMEM or DMSO alone (0.5% final concentration) were used as negative controls in all assays.

### 2.4. Cell Cytotoxicity Assays

Cell cytotoxicity was determined by both MTT and trypan blue assays. Briefly, HeLa, SiHa, CaSki, C33A, and HaCaT cells (5 × 10^4^ cells/mL per well) were inoculated into 96-well plates upon reaching subconfluence (70%–80% confluency) and were treated with 2.5–100 *μ*M apigenin as described above for 24, 48, or 72 h.

After the incubation, MTT (5 mg/mL) was added to each well, and the plates were incubated in the dark at 37°C for 4 h. At the end of the incubation, the medium was removed, the resulting formazan was dissolved in 150 *μ*L DMSO, and the optical density (OD) was measured at 570 nm using a multiwell microplate reader (Bio Tek-Power Wave XS, VT, USA). The 3-(4,5-dimethyl- 2-thiazolyl)-2,5-diphenyl-2H-tetrazolium bromide (MTT) assay is based on the ability of living cells to reduce MTT to insoluble formazan crystal violet via mitochondrial dehydrogenase [49].

For the trypan blue exclusion staining [50], after the incubation period, the medium was removed, the cells were washed with phosphate-buffered saline (PBS), and 200 *μ*L of 0.25% trypsin/EDTA solution was added to detach the cells from the plate. Cell viability was assessed by counting live versus dead cells using standard trypan blue (0.4% in PBS) on a hemocytometer under an inverted microscope (EVOS FL Cell Imaging System, Life Technologies, CA, USA). For both the MTT and trypan blue assays, the results were expressed as a percentage of the control cells, which was considered to represent 100% cell viability. The data are shown as the mean values ± standard deviation (SD) of three independent experiments conducted in triplicate.

IC_50_ (i.e., the concentration that inhibited cell growth by 50% compared to untreated controls) and IC_90_ (i.e., the concentration that inhibited cell growth by 90% compared to untreated controls) values were obtained by nonlinear regression analysis using GraphPad Prism (GraphPad Software, San Diego, CA).

Additionally, each cell line (5 × 10^4^ cells/mL) was inoculated onto 6-well plates and cultured in 10% FBS-DMEM medium at 37°C in a humidified atmosphere with 5% CO_2_. After reaching subconfluence, the cells were treated with 10% FBS-DMEM medium containing 2.5, 60, and 100 *μ*M apigenin. Untreated cells were used as controls. The growth state and morphology of the cells were observed after 72 h under an inverted microscope (EVOS FL Cell Imaging System, Life Technologies, CA, USA).

### 2.5. Colony Forming Assay

To determine the long-term cytotoxicity effects of apigenin, a clonogenic assay was used [51]. All cell lines were seeded in 60 mm plates at a density of 600 cells/plate/4 mL. After 24 h, the cells were exposed to apigenin and incubated in ideal conditions for 14 days (medium was changed every 3 days). Additionally, the recovery ability of colonies was evaluated. For this purpose, we treated the plates with apigenin, which had exhibited toxic effects after 14 days of exposure in previous studies, for 1, 6, 18, 24, 48, or 72 h or 7 days. After each exposure time, supernatants were exchanged for fresh DMEM medium with 10% FBS without dye, and the cell cultures were kept for 14 days. The colonies formed from each cell line were stained with crystal violet after fixation with methanol and counted manually. The results are expressed as survival fractions, which were obtained by dividing the number of colonies that arose after treatment by the number of cells seeded and plate efficiency (PE: number of colonies formed by untreated cells/number of cells seeded) multiplied by 100.

### 2.6. Analysis of Senescence

Cell senescence was evaluated as described by Gary and Kindell (2005) using *β*-galactosidase [52]. Briefly, cell lines were incubated with apigenin (IC_50_ of each cell line) for 48 h before *β*-galactosidase activity determination. Then, the cells were washed twice in PBS and fixed in fixation solution containing 0.5% glutaraldehyde for 15 min. The fixation solution was removed by washing the cells twice with PBS containing MgCl_2_, and then X-Gal staining solution was added. The cells were then incubated at 37°C in a CO_2_-free environment for 4 h. Doxorubicin was used at a concentration of 5 *μ*g/mL as a positive control. Cells with blue-stained cytoplasm were considered senescent, and the percentage of such cells was determined after counting three random fields of 100 cells each. Data are shown as the mean value ± SD of three independent experiments conducted in triplicate.

### 2.7. Analysis of Cell Death by Apoptosis

Apoptosis was assayed using Annexin V-FITC/propidium iodide (PI) based on a previously described protocol with some modification [53]. Briefly, 1.0 × 10^4^ cells/well were seeded overnight in 24-well plates. The cells were treated with apigenin (IC_50_ of each cell line) for 48 h. After treatment, the cells in the supernatant and the adherent cells were washed with PBS and binding buffer (10 mM HEPES, pH 7.5, containing 140 mM NaCl and 2.5 mM CaCl_2_) and stained with 1 *μ*g of FITC-conjugated Annexin-V for 15 min and 40 *μ*g/mL of PI for 5 min. Camptothecin (20 *μ*M) and digitonin (80 *μ*M) were used as positive controls for apoptosis and necrosis, respectively. Cells not treated with apigenin were used as negative controls. Each sample was analyzed using an inverted flow microscope (EVOS FL Cell Imaging System, Life Technologies, CA, USA) to distinguish apoptotic (green fluorescence) and necrotic cells (red fluorescence).

### 2.8. Analysis of Cell Membrane Integrity

Cell membrane integrity was evaluated as described by Britta et al. (2012) with minor modifications, using PI to determine whether cell death triggered by apigenin involved the necrotic death pathway [54]. To accomplish this, cells were treated with apigenin (IC_50_ according to each cell line) for 48 h, washed with PBS, and incubated with 4 *μ*g/mL PI for 10 min. Digitonin (80 *μ*M) was used as a positive control, and untreated cells were used as negative controls. Fluorescence was determined at an excitation wavelength of 480 nm and an emission wavelength of 580 nm under a fluorescence microplate reader (Victor X3, PerkinElmer, Finland). Arbitrary units (relative fluorescence units, RFU) were directly based on fluorescence intensity, and the fluorescence was normalized to the number of cells [55].

### 2.9. Assessment of Cellular Oxidative Stress

Total reactive oxygen species (ROS), mitochondrial transmembrane potential, lipid peroxidation, and extracellular H_2_O_2_ levels were measured using spectrofluorometric assays with a fluorescence microplate reader (Victor X3, PerkinElmer, Finland). Arbitrary units (RFU) were based directly on fluorescence intensity, and the fluorescence was normalized to the number of cells. For all assays, cells not treated with apigenin were used as negative controls.

#### 2.9.1. Measurement of Total Reactive Oxygen Species

Total ROS production was measured based on an increase in fluorescence caused by the conversion of nonfluorescent dye to highly fluorescent 2′,7′-dichlorodihydrofluorescein diacetate (H_2_DCFDA) [56]. Cells (2.5 × 10^5^ cells/mL) were plated in 24-well plates and incubated at 37°C in CO_2_ for 24 h. Then, the cells were incubated with apigenin (IC_50_ according to cell line) for 48 h, centrifuged, washed, and resuspended in PBS (pH 7.4). Afterwards, the cells were treated with 10 *μ*M H_2_DCFDA, a permeable probe, in the dark for 30 min. H_2_O_2_ (200 *µ*M) was used as a positive control. Fluorescence intensity was analyzed at an excitation wavelength of 488 nm and an emission wavelength of 530 nm.

#### 2.9.2. Detection of Extracellular H_2_O_2_ Levels

We assessed the production of H_2_O_2_, a type of ROS, using an Amplex Red assay kit (Molecular Probes, Life Technologies) according to the manufacturer's instructions. Cells (2.5 × 10^5^ cells/mL) were plated in 24-well plates, treated with apigenin (IC_50_ according to each cell line) and incubated for 48 h at 37°C in CO_2_. Following the treatments, trypsinized cells were suspended in PBS containing Amplex Red reagent (12 *μ*M) and horseradish peroxidase (0.05 U/ml). H_2_O_2_ (200 *µ*M) was used as a positive control. Fluorescence was determined at an excitation wavelength of 563 nm and an emission wavelength of 580 nm.

#### 2.9.3. Determination of Mitochondrial Transmembrane Potential (ΔΨ*m*)

Changes in mitochondrial transmembrane potential (ΔΨ*m*) were analyzed using a TMRE (tetramethylrhodamine, ethyl ester) assay [57]. Briefly, cells (2.5 × 10^5^ cells/mL) were seeded in 24-well plates and incubated for 48 h at 37°C in CO_2_. Then, they were treated with apigenin (IC_50_ of each cell line) for 48 h at 37°C in a CO_2_ incubator. Supernatants were removed from the culture dishes, and adherent cells were detached with trypsin-EDTA. The cells were collected by centrifugation, resuspended in staining solution with 25 nM TMRE, and incubated at 37°C in CO_2_ for 30 min in the dark. Carbonyl cyanide m-chlorophenylhydrazone (CCCP) was used as a positive control (100 *µ*M). Fluorescence intensity was analyzed at an excitation wavelength of 540 nm and an emission wavelength of 595 nm.

#### 2.9.4. Lipid Peroxidation Assay

The extent of lipid peroxidation (LPO) was determined based on the amount of diphenyl-1-pyrenylphosphine (DPPP), which is essentially nonfluorescent until it is oxidized to a phosphine oxide (DPPP-O) by peroxides [58]. Cells (2.5 × 10^5^ cells/mL) were plated in 24-well plates and incubated at 37°C in CO_2_. Then, they were treated with apigenin (IC_50_ according to each cell line) for 48 h, followed by treatment with 50 *μ*M DPPP for 15 min at room temperature. Hydrogen peroxide was used as a positive control (200 *µ*M). Fluorescence was determined at an excitation wavelength of 355 nm and an emission wavelength of 380 nm.

#### 2.9.5. Catalase Activity Measurement

We measured the activity of catalase, an enzyme involved in the cell antioxidant system, based on the ability of the enzyme to break down H_2_O_2_. Briefly, cells (5 × 10^5^ cells/mL) were plated in 6-well plates. Then, they were treated with apigenin (IC_50_ and IC_90_ for each cell line) for 48 h at 37°C in CO_2_. Following the treatments, the cells were lysed with RIPA buffer for protein extraction on ice. The lysates were added to 1 M Tris buffer containing 5 mM EDTA and 50 mM H_2_O_2_ (pH 8.0). The rate of H_2_O_2_ decomposition was monitored spectrophotometrically (UV-2550, Shimadzu, Japan) at 240 nm for 60 seconds. Catalase activity was expressed as H_2_O_2_ consumed/min × mg protein (*ε*, 33.33 M^−1^ × cm^−1^) [59].

### 2.10. Cell Migration and Invasion Analysis

#### 2.10.1. Wound-Healing Migration Assay

Wound-healing assays were performed as previously described [60]. Suspensions of each cell line were seeded in 6-well plates (2.5 × 10^4^ cells/mL) and cultured in medium containing 10% FBS. Confluent monolayers of the cells were then mechanically scratched with a blue pipet tip (1000 *μ*L), and cell debris was removed by washing with PBS. Then, the wounded monolayer was incubated with apigenin (IC_30_ treatment), DMSO, and culture medium (controls). Cell migration into the scratched region was recorded using an inverted microscope (EVOS FL Cell Imaging System, Life Technologies, CA, USA) at 0, 24, 48, and 72 h. Wound closure after 24, 48, and 72 h was compared to the initial measurements.

#### 2.10.2. Invasion Assay

Transwell invasion chambers containing polycarbonate filters (8 *μ*m, Costar Corp., Cambridge, MA) were coated on the upper surface with Matrigel (Becton-Dickinson, San Jose, CA). Cervical cancer cell lines (5 × 10^4^ cells/mL) were suspended in serum-free DMEM and added to the upper chamber. Both the lower and upper chambers contained DMEM and apigenin (IC_50_ and IC_90_ of each cell line). Cells not treated with apigenin were used as a negative control. All cells were incubated for 48 h at 37°C in CO_2_. Cells on the upper surface of the filter were completely removed by wiping them with a cotton swab. Cells that had invaded through the Matrigel and reached the lower surface of the filter were fixed in 10% formalin, stained with a toluidine blue solution, and counted under a light microscope at 20x magnification. The mean number of cells in 10 fields was calculated, and the assay was performed in triplicate [61].

### 2.11. Statistical Analysis

Data distributions were expressed as mean ± standard deviation (SD) of independent experiments in triplicate. Significant differences among means were identified using the GraphPad Prism® 6.0 software (CA, USA). The ANOVA test followed by Tukey–Kramer test was used to calculate the multiple comparisons, for example, cell death by apoptosis, cell membrane integrity, cellular oxidative stress, reactive oxygen species, cells migration, and invasion analysis. The Student's *t*-distribution was used to compare cytotoxic effects of apigenin on cervical cancer cell lines to control cells. Values of *P* < 0.05 were considered statistically significant.

## 3. Results

### 3.1. Apigenin Inhibits Cervical Cancer Cell Viability but Is Not Cytotoxic to HaCaT Cells

To study the effects of apigenin treatment on tumor cells as well as normal cells, we exposed four cervical cancer cell lines, the HeLa (integrated HPV 18), SiHa (integrated HPV 16), CaSki (integrated HPV 16 and HPV 18), and C33A (without HPV) cell lines, as well as a human immortalized keratinocyte (HaCaT) cell line (control cells), to increasing doses of apigenin over a maximum of 72 h.

**Figure 2:**
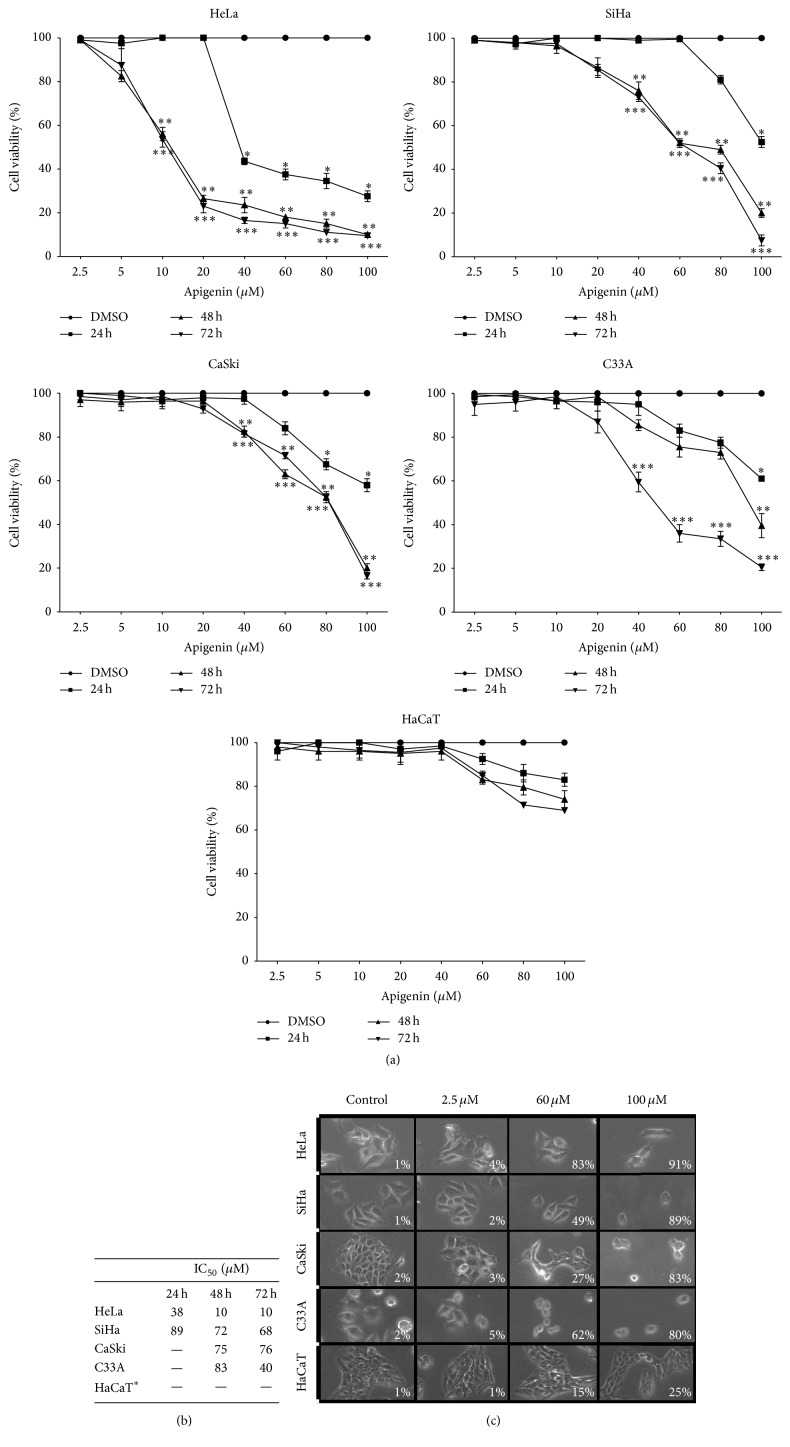
Cytotoxic effects of apigenin on cervical cancer cell lines (HeLa, SiHa, CaSki, and C33A cells) and a human keratinocyte cell line (HaCaT cells). (a) Dose-response curves indicating the viability of the cervical cancer cell lines and the HaCaT cells (control cells) following exposure to apigenin (2.5–100 *μ*M) for 24, 48, and 72 h. A statistically significant difference in cell viability was observed between the HeLa, SiHa, CaSki, and C33A cells and the HaCaT cells. (^*∗*^), (^*∗∗*^), and (^*∗∗∗*^) represent statistically significant (*P* < 0.05) differences (24, 48, and 72 h, resp.) between the cancer cell lines and the control cells. (b) Approximate IC_50_ values determined according to the cell viability obtained in (a). ^*∗*^For the HaCaT cells, the IC_50_ value was > 100 *µ*M. Each line represents the mean ± SD of three separate experiments conducted in triplicate. (c) Differential effects on cell morphology induced by apigenin after 72 h of exposure. Cell photomicrographs were taken (20x magnification), and the percentage of cell death was determined by Trypan blue staining. Note that the HaCaT cells do not show morphological changes.

As indicated in Figures 2(a)–2(c), apigenin exerted concentration-dependent cytotoxic effects on all cervical cancer cell lines tested, with an IC_50_ of 10 *μ*M for HeLa, 68 *μ*M for SiHa, 76 *μ*M for CaSki, and 40 *μ*M for C33A cells at 72 h. Apigenin showed selective action in cancer cells, as it was not able to significantly reduce HaCaT cell viability at the tested concentrations. The IC_50_ values are shown in Figure 2(b).

The dose-response graph obtained from the MTT assays and trypan blue dye exclusion tests shows a significant decrease in the percentage of cell viability of all cervical cancer cell lines compared to the HaCaT cells after apigenin exposure (Figure 2(a)). More specifically, among the cervical cancer cell lines tested, the HeLa cells showed a higher reduction of cell viability at a lower apigenin concentration (10 *μ*M) at 48 h (*P* = 0.0012) and 72 h (*P* = 0.001) of exposure. The CaSki and SiHa cell lines showed similar decreases in cell viability, reaching significant levels at 40 *μ*M at 48 h (*P* = 0.029 and *P* = 0.017, resp.) and at 72 h (*P* = 0.012 and *P* = 0.008, resp.). Additionally, the C33A cell line showed a significant reduction in cell viability at 40 *μ*M (*P* = 0.021), but only after 72 h of apigenin exposure. Additionally, apigenin did not significantly reduce HaCaT cell viability at any concentration or time tested (*P* = 0.321), highlighting the selective action of apigenin towards cancer cells.

The cell growth inhibition induced by apigenin was further verified by microscopic observation. The results in Figure 2(c) show that the growth of HeLa, SiHa, CaSki, and C33A cells was effectively inhibited after exposure to 2.5–100 *μ*M apigenin for 72 h, whereas HaCaT cell growth was unaffected. Apigenin also induced pronounced morphological changes due to cell death when the cervical cancer cell lines were exposed to 60 *μ*M and 100 *μ*M concentrations for 72 h. The cells exhibited retraction of cytoplasmic expansion and detachment from the plate due to cell death. Morphological changes were not observed in HaCaT cells exposed to the same concentrations of apigenin for the same length of time.

**Figure 3:**
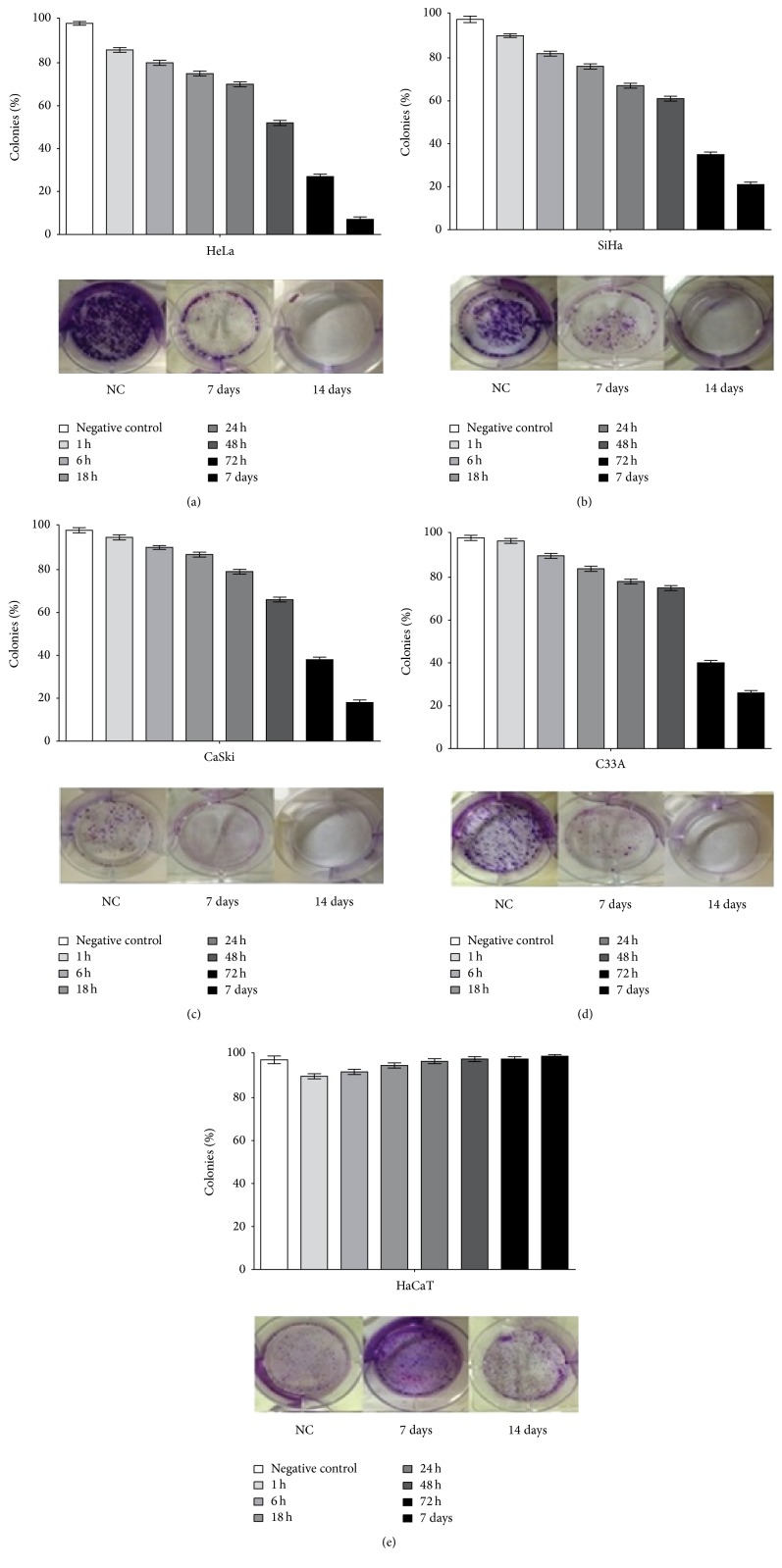
The effect of apigenin exposure on the clonogenicity of cervical cancer cell lines and human keratinocytes at 1, 6, 18, 24, and 48 h and 7 and 14 days followed by culture with DMEM. The graph indicates that colony recovery diminished with increasing times of exposure in cervical cancer cell lines ((a) HeLa; (b) SiHa; (c) CaSki; (d) C33A) and increased with time of exposure in HaCaT cells (e). Data are shown as the mean values ± SD of three independent experiments conducted in triplicate. Photos indicate that exposure to apigenin reduced colony formation by 7 days and prevented colony formation after 14 days in HeLa (a), SiHa (b), CaSki (c), and C33A (d) cells. In HaCaT cells (f), colony formation continued to be equivalent after 7 and 14 days of apigenin exposure.

To further examine the long-term cytotoxicity of apigenin, clonogenic assays were performed. For this purpose, we exposed all cervical cancer cell lines and HaCaT cells to subtoxic doses of apigenin (IC_30_). After 14 days of incubation, colony formation was inhibited by 100% in the HeLa, SiHa, CaSki, and C33A cells compared with untreated cells (Figure 3). In the HaCaT cells, colony formation was equivalent after 14 days of incubation with apigenin compared to the untreated cells. Based on this finding, we tested the capability of the cells to recover from damage after 1, 6, 18, 24, 48, and 72 h and 7 days of exposure to apigenin, followed by the addition of DMEM. The results presented in Figure 3 indicate that although recovery occurred after 1 h of exposure, there was a decrease in the number of colonies after this time.

These data indicate that apigenin exerts concentration-dependent cytotoxic effects on HeLa, SiHa, CaSki, and C33A cells. More importantly, apigenin showed selective action towards cancer cells, as it did not reduce HaCaT viability.

### 3.2. Apigenin Does Not Induce Cell Senescence

Cell senescence was studied by staining [52] with *β*-galactosidase, a biomarker for senescence in mammalian cells, which exhibit lysosomal-galactosidase activity at an optimal pH of 4.0. Cells that are in a state of replicative senescence express senescence-associated galactosidase activity, which is measured at pH 6.0. Senescence was not observed after apigenin exposure in any cervical cancer cell line or in the HaCaT cells, as blue SA-b-Gal staining was not observed. Therefore, we found that apigenin did not effectively induce senescence in cervical cancer cells (data not shown).

### 3.3. Apigenin Induces Apoptotic Death in Cervical Cancer Cells

**Figure 4:**
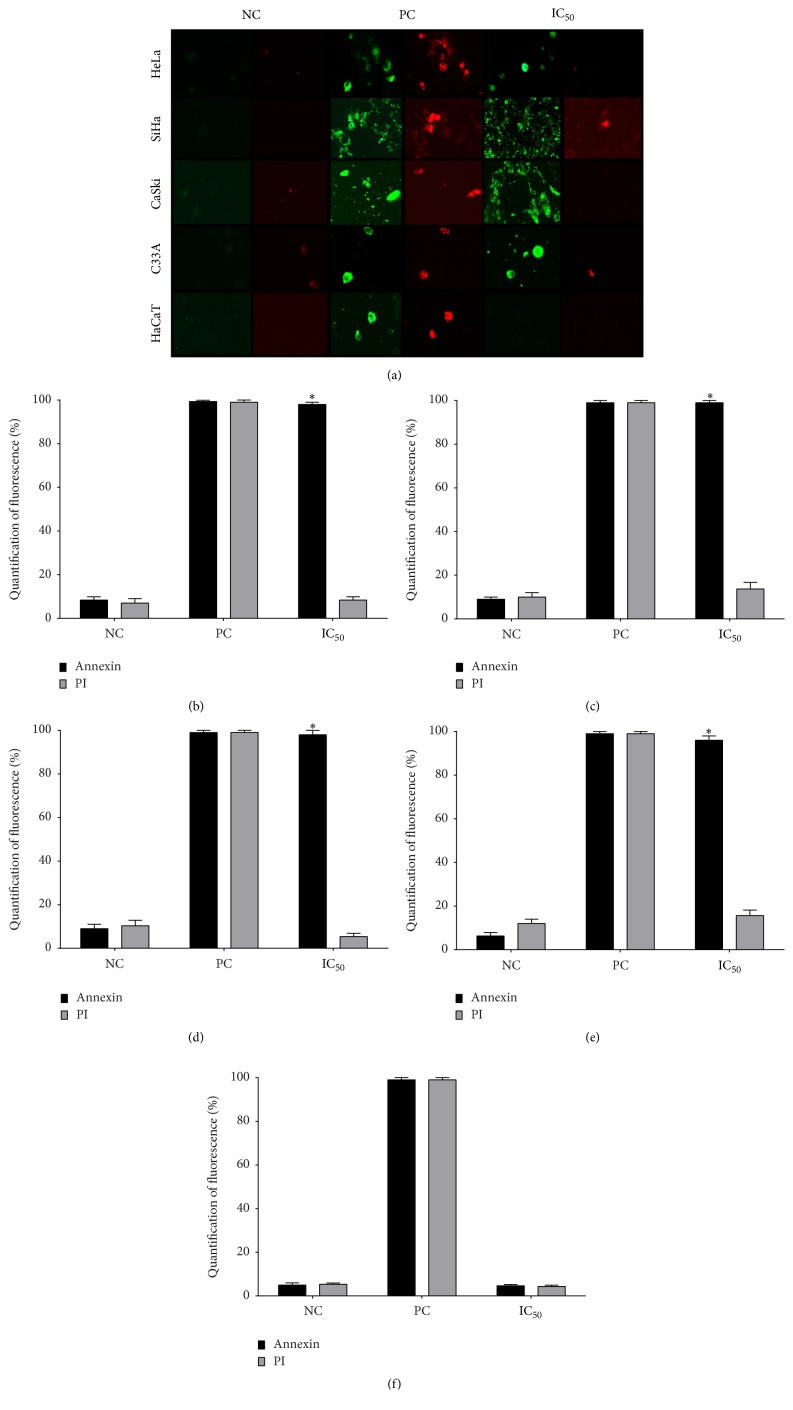
Assessment of death pathway associated with apigenin in cervical cancer cell lines and HaCaT cells. (a) Representative figures of cancer cell lines (HeLa, SiHa, CaSki, and C33A) and HaCaT cells exposed to apigenin (IC_50_ of each cell line) at 48 h stained with the apoptosis marker Annexin V (green fluorescence) and the necrosis marker propidium iodide (PI) (red fluorescence). (b, c, d, e, and f) Histograms show the mean % Annexin V-positive cells (HeLa, SiHa, CaSki, C33A, and HaCaT cells, resp.) treated with apigenin (IC_50_ of each cancer cell line) for 48 h. Camptothecin (20 *μ*M) and digitonin (80 *μ*M) were used as positive controls for apoptosis and necrosis, respectively (PC), and cells not treated with apigenin were used as negative controls (NC). Data are shown as the mean ± SD of three independent experiments conducted in triplicate. ^*∗*^*P* < 0.05 versus the negative control. Magnification: 20x.

As described above, apigenin treatment induces a significant decrease in cancer cell viability. To determine the type and extent of cell death, we analyzed whether apigenin could induce apoptosis in cervical cancer cells via an Annexin V-FITC/PI assay using fluorescence imaging. Annexin V staining detects the translocation of phosphatidylserine from the inner to the outer cell membrane during early apoptosis (green fluorescence), and PI can enter the cell during necrosis or late-stage apoptosis; it can also enter dead cells (red fluorescence) [53]. Apigenin induced apoptosis in all cervical cancer cell lines after 48 h of exposure. As shown in Figure 4(a), cellular apoptosis considerably increased in the apigenin-treated cancer cells compared to the control group. More specifically, at the IC_50_ of each cancer cell line, apigenin induced significant apoptosis, as the cells were positively marked with Annexin V (green) but not induced to undergo necrosis (unmarked PI-red). Apigenin did not induce death in HaCaT cells (unmarked by either Annexin V or PI).

In Figures 4(b)–4(f), the histograms show the mean % Annexin V-positive cells in the cell lines treated with apigenin (IC_50_ of each cancer cell line) for 48 h. Mean Annexin V-positive cell numbers of approximately 100% were found in the HeLa (*P* = 0.0001; Figure 4(b)), SiHa (*P* = 0.00015; Figure 4(c)), CaSki (*P* = 0.00012; Figure 4(d)), and C33A (*P* = 0.00016; Figure 3(e)) cells, whereas approximately 5–15% of cells were PI-positive. In Figure 4(f), the histogram shows that apigenin exposure for 48 h did not induce death in HaCaT cells; only approximately 4% of these cells were marked with Annexin V (*P* = 0.2879) and PI, similar to the negative control. These data demonstrate that apigenin can selectively induce apoptosis in cervical cancer cells.

**Figure 5:**
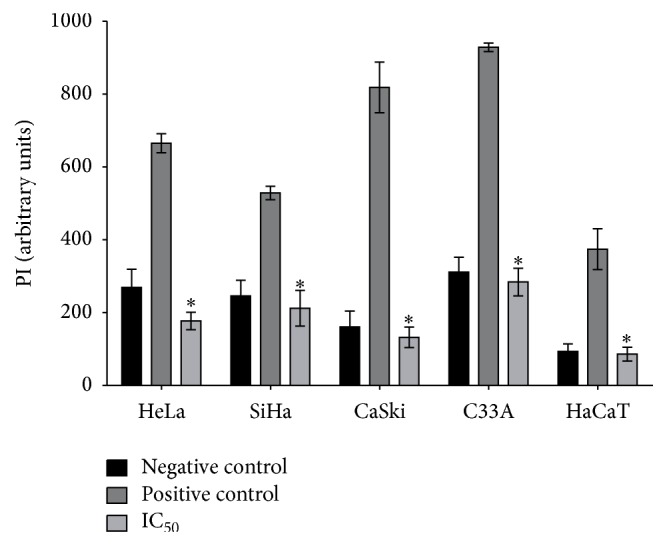
Effects of apigenin on cell membrane integrity in cervical cancer cell lines and HaCaT cells. HeLa, SiHa, CaSki, C33A, and HaCaT cells were exposed to apigenin (IC_50_ of each cell line), and cell membrane integrity was detected using a PI fluorescence probe. Arbitrary units (relative fluorescence units, RFU) were based directly on fluorescence intensity. Data are expressed as the mean fluorescence (in arbitrary units) ± SD of three independent experiments conducted in triplicate. ^*∗*^*P* < 0.05 versus the positive control.

To further confirm the mechanism of cell death triggered by apigenin, we evaluated plasma membrane integrity in cervical cancer cell lines and HaCaT cells treated with apigenin and stained with PI, which diffuses across permeable membranes and binds to nucleic acids [54]. As shown in Figure 5, all cervical cancer cell lines showed significantly lower fluorescence than the positive control (HeLa, *P* = 0.011; SiHa, *P* = 0.024; CaSki, *P* = 0.001; C33A, *P* = 0.0013) and HaCaT cells (*P* = 0.0112) after apigenin exposure (IC_50_). These data indicate that apigenin exposure did not induce the cell membrane rupture that occurs in necrosis and late apoptosis and confirm that apoptosis is the death pathway triggered by apigenin.

### 3.4. Apigenin Induces Oxidative Stress in Cervical Cancer Cell Lines

**Figure 6:**
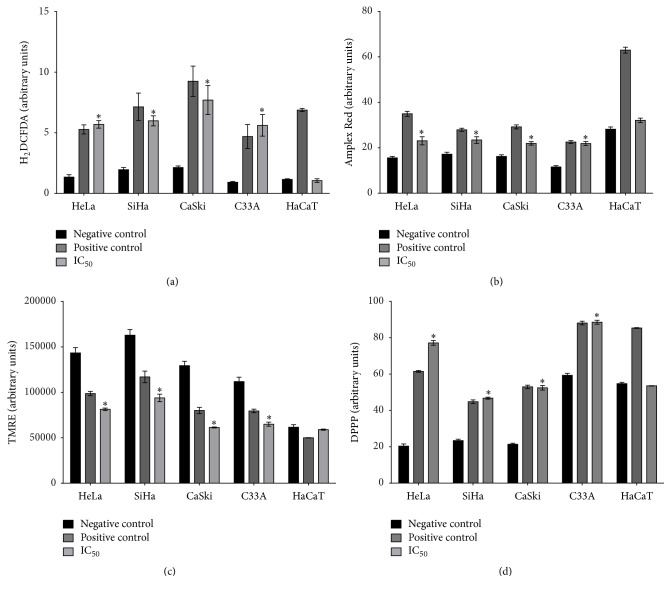
Effects of apigenin on cellular oxidative stress. (a) Total ROS production was evaluated during exposure of cervical cancer cell lines (HeLa, SiHa, CaSki, and C33A) and HaCaT cells to apigenin using the fluorescence probe H_2_DCFDA. The positive control was treated with 10 *μ*M H_2_DCFDA in the dark. (b) Detection of extracellular H_2_O_2_ levels in all cell lines exposed to apigenin was conducted using an Amplex® Red assay kit. H_2_O_2 _was used as a positive control. (c) Mitochondrial membrane potential (Δ*ψm*) after exposure to apigenin measured using a TMRE fluorescence probe. Carbonyl cyanide m-chlorophenylhydrazone (CCCP) was used as a positive control. (d) Lipid peroxidation (LP) after exposure to apigenin using the fluorescence probe diphenyl-1-pyrenylphosphine (DPPP). Hydrogen peroxide was used as a positive control. Arbitrary units (relative fluorescence units, RFU) were based directly on fluorescence intensity. Data are expressed as the mean ± SD of three independent experiments conducted in triplicate. ^*∗*^*P* < 0.05 versus negative control.

We next investigated oxidative stress because of the high antioxidant potential attributed to apigenin [14, 62]. We began studying the mechanistic action of this compound by examining the production of total ROS. To accomplish this, we evaluated the effects of total ROS production after apigenin exposure in the cervical cancer cell lines and HaCaT cells using H_2_DCFDA, a fluorescent probe. This probe primarily detects H_2_O_2_ and hydroxyl radicals and fluoresces after forming dichlorofluorescein [63]. Our results showed that apigenin significantly increased total ROS production in all cervical cancer cell lines compared with the negative control (untreated cells) (HeLa, *P* = 0.013; SiHa, *P* = 0.015; CaSki, *P* = 0.0021; C33A, *P* = 0.011). This increase in ROS production was similar to that induced by the positive control (cells treated only with H_2_DCFDA). Moreover, total ROS production was not changed in the HaCaT cells after exposure to apigenin (*P* = 0.214); rather, the cells maintained ROS levels similar to the negative control (Figure 6(a)). Because increased ROS generation in the cytosol occurs in most apoptotic cells, these results further support that apoptosis is the cell death pathway caused by apigenin and that this is likely a result of oxidative stress.

Next, we assessed the production of H_2_O_2_, which is a type of ROS. Extracellular H_2_O_2_ levels were detected using an Amplex Red assay. Our results showed that apigenin significantly increased H_2_O_2_ levels in all cervical cancer cell lines compared with the negative control (untreated cells) (HeLa, *P* = 0.0164; SiHa, *P* = 0.0212; CaSki, *P* = 0.0055; C33A, *P* = 0.0005). H_2_O_2_ production was not changed after exposure to apigenin in HaCaT cells (*P* = 0.0506) (Figure 6(b)).

We next evaluated the effect of apigenin exposure on mitochondrial membrane potential (ΔΨ*m*). Δ*ψm* changes are an additional indication of apoptosis; Δ*ψm* contributes to the process that facilitates the exit of many apoptogenic factors to the cytosol. We used a TMRE assay, which quantifies changes in mitochondrial membrane potential in live cells, and a cell-permeable, positively charged, red-orange dye that readily accumulates in active mitochondria due to their relative negative charge. Depolarized or inactive mitochondria exhibit decreased Δ*ψm* and failure to sequester TMRE [57]. Our results showed that apigenin significantly decreased the Δ*ψm* in all cervical cancer cell lines compared with the negative control (untreated cells) (HeLa, *P* = 0.0032; SiHa, *P* = 0.0393; CaSki, *P* = 0.0055; C33A, *P* = 0.0081). Furthermore, Δ*ψm* did not change in HaCaT cells after exposure to apigenin (*P* = 0.0668) (Figure 6(c)).

Next, we evaluated the effect of apigenin exposure on lipid peroxidation (LPO), which can be defined as a cascade of biochemical events resulting from the action of free radicals on the unsaturated lipids of cell membranes. This process primarily generates alkyl, peroxyl, and alkoxyl radicals, leading to the destruction of unsaturated lipid structure, the failure of mechanisms that exchange metabolites, and the induction of cell death by apoptosis. Therefore, LPO can be used as an indicator of cellular oxidative stress [58]. We determined the amount of diphenyl-1-pyrenylphosphine (DPPP) that is essentially nonfluorescent until it is oxidized to a phosphine oxide (DPPP-O) by peroxides. Our results showed that apigenin significantly increased LPO in all cervical cancer cell lines compared with the negative control (untreated cells) (HeLa, *P* = 0.0001; SiHa, *P* = 0.001; CaSki, *P* = 0.0008; C33A *P* = 0.0022). LPO was not changed in HaCaT cells after exposure to apigenin (*P* = 0.1934) (Figure 6(d)).

**Figure 7:**
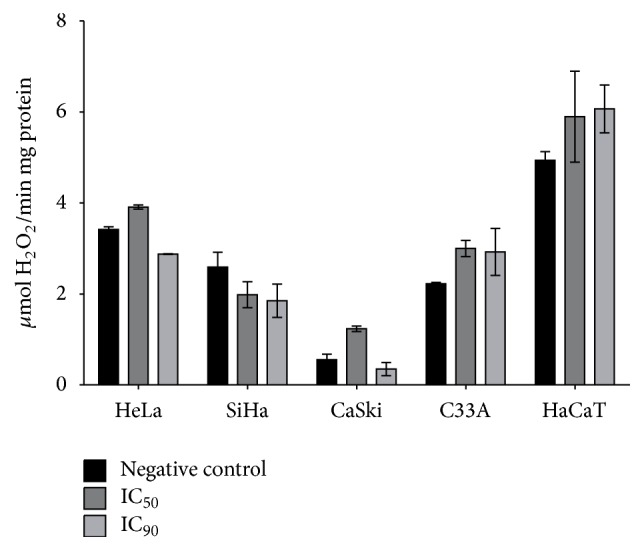
Effects of apigenin on catalase activity after exposure to apigenin (IC_50_ and IC_90_ of each cell line) for 48 h based on H_2_O_2_ consumed. Catalase activity was expressed as H_2_O_2_ consumed/min × mg protein (*ε*, 33.33 M^−1^ × cm^−1^).

Finally, we measured the activity of catalase, an enzyme involved in the cell antioxidant system that is responsible for maintaining low levels of ROS and cell homeostasis. As shown in Figure 7, the catalase activity in HaCaT cells gradually increased after apigenin exposure (IC_50_ and IC_90_, resp.). In HeLa, CaSki, and C33A cells, an increase in enzyme activity occurred following exposure to the IC_50_ of apigenin, whereas reduced activity was observed following exposure to the IC_90_. In contrast, SiHa cells exhibited reduced catalase activity after exposure to the IC_50_ of apigenin, and an even greater reduction was observed following exposure to the IC_90_.

### 3.5. Apigenin Inhibits Cervical Cancer Cell Migration and Invasion

**Figure 8:**
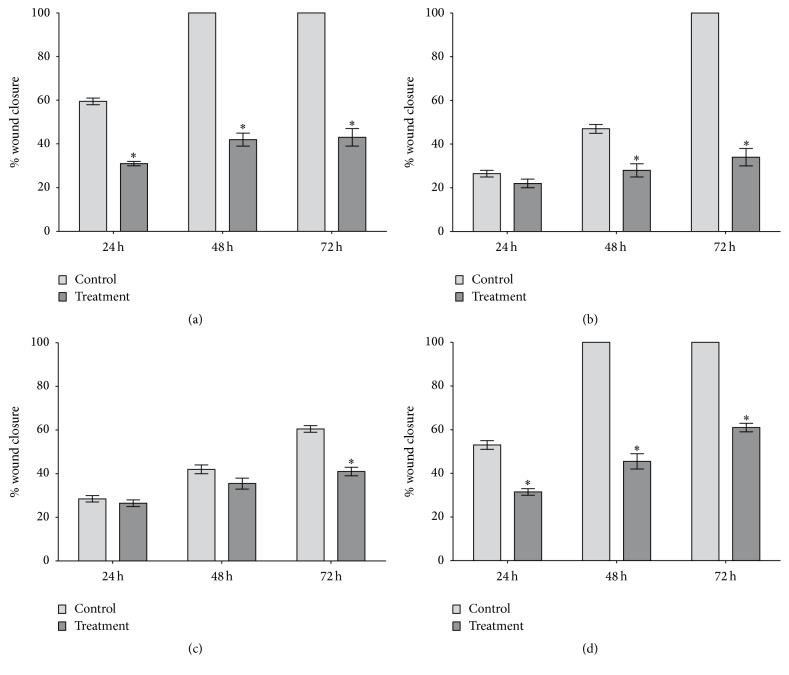
Cell migration analysis using a wound-healing assay. HeLa (a), SiHa (b), CaSki (c), and C33A (d) cells were tested in 6-well plates (2.5 × 10^4^ cells/well) after scratching in the absence (negative control) and presence of apigenin. The results were calculated by comparing wound closure after 24, 48, and 72 h with the measurements taken at the initial time, and data are shown as the mean ± SD of three independent experiments conducted in triplicate. ^*∗*^*P* < 0.05 versus the negative control.

The wound-healing assay revealed that apigenin (IC_30_) effectively inhibited cell migration in all cancer cell lines studied; the greatest reduction in basal migratory ability was for HeLa cells, followed by C33A, SiHa, and CaSki cells. For the C33A and HeLa cells, apigenin significantly inhibited cell migration by up to twofold at all times tested (*P* = 0.0059 and *P* = 0.029, resp.). Additionally, apigenin inhibited CaSki and SiHa cell migration at later exposure times. For the SiHa cells, inhibition was higher than in all other cancer cell lines analyzed (up to threefold) at 72 h (*P* = 0.0038). Finally, the CaSki cells showed significant inhibition of migration (approximately twofold) only at 72 h of apigenin exposure (*P* = 0.016) (Figure 8).

**Figure 9:**
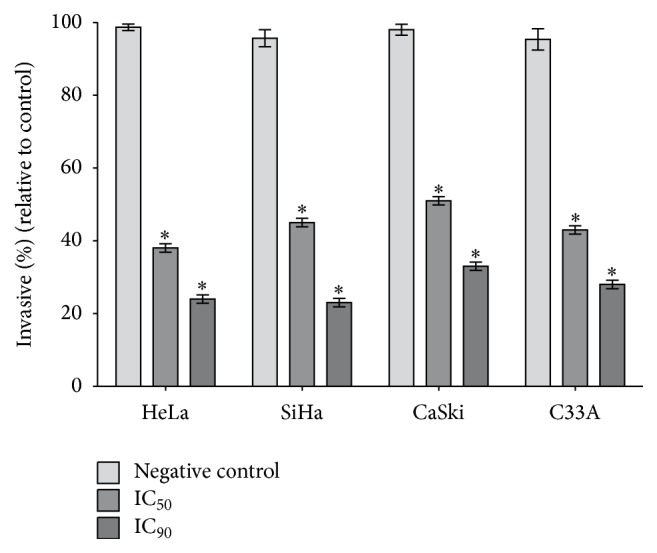
Effects of apigenin on cell invasion. Cervical cancer cell lines (HeLa, SiHa, CaSki, and C33A) were seeded onto Matrigel-coated filters in Boyden chambers. After 48 h of apigenin treatment (IC_50_ and IC_90_ of each cell line), the number of cells present on the bottom side of the filter was quantified and expressed as a percentage of the negative control. The values are presented as the mean ± SD of three independent experiments conducted in triplicate. ^*∗*^*P* < 0.05 versus the negative control.

Invasion ability was measured by the number of cells that migrated through a reconstituted Matrigel layer to the bottom surface of a porous membrane in a Transwell chamber assay. As shown in Figure 9, both concentrations of apigenin (IC_50_ and IC_90_ of each cell line) reduced the number of cells in the bottom surface of the Transwell chamber, indicating a decrease in the invasiveness of all four cell lines. There was a further significant reduction in cell invasion observed at the IC_50_ and IC_90_ of apigenin in the HeLa (*P* = 0.0018 and *P* = 0.0008, resp.), SiHa (*P* = 0.0021 and *P* = 0.0005, resp.), CaSki (*P* = 0.0025 and *P* = 0.0012, resp.), and C33A (*P* = 0.0015 and *P* = 0.0001, resp.) cells compared to the control cells.

## 4. Discussion

In the present study, we evaluated the cytotoxic effects of apigenin in a comprehensive panel of human cervical cancer-derived cell lines, including HeLa (HPV 18-positive), SiHa (HPV 16-positive), CaSki (HPV 16 and 18-positive), and C33A (HPV-negative) cells, compared to a nontumorigenic spontaneously immortalized human epithelial cell line (HaCaT). The results demonstrated that apigenin exposure had a selective time- and dose-dependent cytotoxic effect in all cervical cancer cell lines, but not in HaCaT cells. Apigenin induced cancer cell death via apoptosis, which was triggered by mitochondrial redox impairment. Additionally, apigenin inhibited cancer cell migration and invasion.

Only three previous studies have evaluated the activity of apigenin specifically on cervical cancer cells; all showed a cytotoxic effect on HeLa cells [39–41]. Our results are in agreement with these reports and highlight additional important information, particularly that apigenin has a significant time- and dose-dependent cytotoxic effect on non-HeLa human cervical cancer-derived cell lines, including SiHa, CaSki, and C33A cells. Apigenin exerted cytotoxic effects on HeLa cells (IC_50_ of 10 *μ*M), SiHa cells (IC_50_ of 68 *μ*M), CaSki cells (IC_50_ of 76 *μ*M), and C33A cells (IC_50_ of 40 *μ*M) at 72 h. Among these cell lines, the HeLa cells showed the greatest reduction in cell viability at the lowest apigenin concentration (10 *μ*M) after 48 h and 72 h of exposure. The CaSki and SiHa cells showed similar decreases in cell viability, reaching significant levels at 40 *μ*M apigenin at 48 h and 72 h. The C33A cells showed a significant reduction in cell viability at 40 *μ*M apigenin, but only after 72 h of exposure. Thus, apigenin showed cytotoxic effects at the lowest exposure concentration and time in cancer cell lines immortalized by HPV (HeLa followed by SiHa and CaSki cells), indicating its potential for the treatment of cervical cancers caused by HPV 16 and HPV 18, which account for approximately 70% of cases [1–4].

Regarding the cytotoxic effect of apigenin on HeLa cells, our study showed lower IC_50_ values than others, with reported IC_50_ values of 40 *μ*M at 30 h [39] and 37 *μ*M at 24 h [41]. We have knowledge of only one study that investigated the cytotoxic activity of apigenin on SiHa and C33A cells [64] that used the same concentrations and times used here (0–100 *μ*M at 72 h). The results from that study are similar to ours: apigenin was cytotoxic for both SiHa and C33A cells with IC_50_ values of 50.14 *μ*M and 45.10 *μ*M, respectively. Likewise, only one study evaluated CaSki cell cytotoxicity after apigenin exposure. In the referenced study, 20 *μ*M apigenin was applied for 24 h, and unlike our results, a cytotoxic effect of apigenin on CaSki cells was not detected [65].

In contrast to its cytotoxic effects on cancer cells, we showed that apigenin did not significantly reduce HaCaT cell viability at any concentration or time tested. These data highlight the selective effect of apigenin towards cancer cells, similar to studies of other human cancer types that reported its low intrinsic toxicity and differential effects in normal versus cancer cells [21–23]. We also evaluated the cytotoxic effect of apigenin using microscopy and found that apigenin induced pronounced morphological changes in all cervical cancer cell lines tested; in all cases, retraction of cytoplasmic expansion and detachment from the plate due to cell death occurred. Finally, we examined the long-term cytotoxicity of apigenin using a clonogenic assay. We found that subtoxic doses of apigenin resulted in 100% inhibition of colony formation in HeLa, SiHa, CaSki, and C33A cells compared with untreated cells. In contrast, HaCaT cells maintained colony formation after apigenin exposure. Based on these findings, we tested the capability of the cells to recover from damage after 1, 6, 18, 24, 48, and 72 h and 7 days of exposure to apigenin. The results indicated that although recovery occurred after 1 h of exposure, there was a decrease in the number of colonies after that time. Overall, these results show that apigenin decreased colony formation at subtoxic doses and had a strong and selective cytotoxic effect on cervical cancer cells immortalized by HPV 16, HPV 18, and HPV 16 and 18 together, as well as on cells not induced by HPV.

To determine the cell death pathway that results from apigenin exposure, we studied senescence with blue SA-b-Gal staining. Cellular senescence is believed to represent a natural cellular process to suppress tumor formation [66]. Our results did not show that apigenin caused cell death by inducing senescence. Next, we evaluated cell death by apoptosis and necrosis by staining with fluorescent Annexin V (detects early apoptosis) and PI (detects necrosis or late apoptosis). We found that apigenin induced death by apoptosis in all cervical cancer cell lines after 48 h exposure. To further support these results, we evaluated membrane integrity by staining with fluorescent PI, which diffuses through permeable membranes and binds to nucleic acids in necrotic cells. Our data indicate that apigenin exposure did not induce the cell membrane rupture that occurs in necrosis and late apoptosis. Together, these data indicate that apigenin exposure induces cancer cell death via apoptosis, in agreement with previous studies of different cancer cell lines, including HeLa cells [40, 41, 66].

To develop effective chemopreventive and chemotherapeutic approaches for target organ carcinogenesis, a promising strategy is to take advantage of the biochemical differences that exist between cancer cells and their normal counterparts. In this respect, agents capable of inducing selective apoptosis of cancer cells are receiving considerable attention as novel cancer-prevention options [67, 68]. The intrinsic pathway of apoptosis is related to oxidative stress, a condition in which there is an imbalance between ROS production and detoxification [69]. ROS play a role in regulating the intrinsic pathway of apoptosis and are associated with reduced mitochondrial membrane potential [70–73]. We investigated whether apigenin exposure induces oxidative stress in cervical cancer cells using assays to detect total ROS and H_2_O_2_ production, changes in mitochondrial membrane potential (ΔΨ*m*), lipid peroxidation (LPO) levels, and catalase activity. We detected significantly increased production of total ROS and extracellular H_2_O_2_, increased LPO levels, and significantly decreased Δ*ψm* and catalase activity in HeLa, SiHa, CaSki, and C33A cells, but not in HaCaT cells. Collectively, our data provide evidence that apigenin induces oxidative stress, which leads to cervical cancer cell death via the intrinsic pathway of apoptosis. Our results are in accordance with previous studies reporting that apigenin can induce the following: (1) increased ROS production in various cancers, including skin [25], colon [66], and lung [74] cancer, as well as in HeLa cells [75, 76]; (2) apoptosis mediated by increased ROS production in human colorectal cancer cells [66]; (3) increased H_2_O_2_ production in the K562 human myelogenous leukemia cell line [77]; and (4) mitochondrial membrane potential collapse in choriocarcinoma cells [78]. In relation to catalase activity, mechanistic studies of compounds that induce apoptotic cell death via ROS have demonstrated the importance of catalase activity inhibition on the accumulation of ROS and consequently cell death by apoptosis [79]. Our evidence that catalase activity was reduced in cancer cell lines after apigenin exposure is compatible with the mechanism of action of apoptosis induction. Likewise, our evidence of increased LPO levels is in accordance with the theory that LPO is a toxic free radical produced by ROS and an indicator of cellular oxidative stress [80].

Still, our results of mitochondrial stress and apoptotic death after exposure to apigenin are in agreement with others in relation to structure activity of these flavone. The activity of apigenin was associated with hydroxyl group on its B ring in position 4′ and its ability to react with free radicals. More specifically, apigenin and kaempferol exhibit similar B ring structure but the presence of a hydroxyl group in position 3 (kaempferol) decrease its cytotoxicity in HeLa cells compared to apigenin. The higher activity of apigenin compared to kaempferol in this study was at least partially dependent on its ability to react with ROS that are important to induction of apoptosis [81].

Regarding the effect of apigenin on the migration and invasion of human cervical cancer-derived cell lines, Czyz et al. (2005) showed that apigenin inhibited cell motility, which correlated with reduced invasive potential of HeLa cells after exposure to 30 and 50 *μ*M apigenin for 24 h [40]. Noh et al. (2010) reported that 5 *μ*M apigenin inhibited the in vitro invasion of CaSki cells [65]. Other studies of choriocarcinoma [78], colorectal cancer [76, 82] and prostate cancer [14] also showed that apigenin suppresses cell migration and invasion. Our results from the wound-healing assays and Matrigel migration and invasion assays showed that, similar to the above-mentioned studies, apigenin inhibited cancer cell migration, with the greatest reduction in basal migratory ability observed in HeLa cells, followed by C33A, SiHa, and CaSki cells, and also significantly decreased cell motility and invasion. These data suggest that apigenin exerts antitumorigenic effects not only by influencing cervical cancer cell cytotoxicity but also by affecting cell motility and thus invasion. This evidence is consistent with the large number of mechanisms that has been attributed to apigenin, including antioxidant properties and their influence on gene expression. The most common activity noted for majority of plant flavones include their role as potent antioxidants and free radical scavengers, with their biological activities related to anti-inflammatory, antimicrobial, antiviral, antimutagenic, and anticancer functions. These biological activities are considered to be related to their interactions with several enzymes and proteins, including calcium phospholipid-dependent protein kinase, DNA topoisomerases, tyrosine protein kinase, phosphorylase kinase, phosphatidylinositol 3-kinase, cytochrome 1A1 expression, and the total cellular glutathione level [83–85].

In conclusion, we found that apigenin has a selective dose-dependent cytotoxic effect and could induce apoptosis in HeLa, SiHa, CaSki, and C33A cells, but not in HaCaT cells. Additionally, apigenin induced mitochondrial redox impairment and inhibited cancer cell migration and invasion. These results show that apigenin had a strong and selective antitumoral effect on cervical cancer cells immortalized by HPV 16, HPV 18, and HPV 16 and 18 together, indicating its potential to be a powerful candidate in developing therapeutic agent for all cervical cancer types. Thus, our data support additional preclinical and clinical studies for further validation of antitumor effects of apigenin applicable to cancer cervical in the future.
